# Impact of Microscopically Positive (≤1 mm) Distal Margins on Disease Recurrence in Rectal Cancer Treated by Neoadjuvant Chemoradiotherapy

**DOI:** 10.3390/cancers15061828

**Published:** 2023-03-17

**Authors:** Luca Sorrentino, Annaclara Sileo, Elena Daveri, Luigi Battaglia, Marcello Guaglio, Giovanni Centonze, Giovanna Sabella, Filippo Patti, Sergio Villa, Massimo Milione, Filiberto Belli, Maurizio Cosimelli

**Affiliations:** 1 Colorectal Surgery Unit, Fondazione IRCCS Istituto Nazionale dei Tumori, 20133 Milan, Italy; 2 Immunotherapy of Human Tumors Unit, Fondazione IRCCS Istituto Nazionale dei Tumori, 20133 Milan, Italy; 3 1st Pathology Division, Department of Pathology and Laboratory Medicine, Fondazione IRCCS Istituto Nazionale dei Tumori, 20133 Milan, Italy; 4 Radiation Oncology Unit, Fondazione IRCCS Istituto Nazionale dei Tumori, 20133 Milan, Italy

**Keywords:** rectal cancer, neoadjuvant chemoradiation, distal margin, locoregional recurrence-free survival

## Abstract

**Simple Summary:**

The adequate distal resection margin in rectal cancer patients after neoadjuvant chemoradiotherapy might be tighter than expected. Patients with a distal margin of ≤1 mm vs. >1 mm were compared: while a distal margin of ≤1 mm may be sufficient in case of major/complete response, a margin of >1 mm is still required to avoid locoregional recurrence in patients with a low response to neoadjuvant treatment. The findings of the present study might also increase the rate of sphincter-preserving rectal surgery in patients with a close/microscopically positive distal margin and a major/complete response to neoadjuvant chemoradiotherapy.

**Abstract:**

Background: The adequate distal resection margin is still controversial in rectal cancer treated by neoadjuvant chemoradiotherapy (nCRT). The aim of this study was to assess the impact of a distal margin of ≤1 mm on locoregional recurrence-free survival (LRRFS). Methods: Among 255 patients treated with nCRT and surgery at the National Cancer Institute of Milan, 83 (32.5%) had a distal margin of ≤1 mm and 172 (67.5%) had a distal margin of >1 mm. Survival analyses were performed to assess the impact of distal margin on 5-year LRRFS, as well as Cox survival analysis. The role of distal margin on survival was analyzed according to different tumor regression grades (TRGs). Results: The overall 5-year LRRFS rate was 77.6% with a distal margin of ≤1 mm vs. 88.3% with a distal margin of >1 mm (Log-rank *p* = 0.09). Only stage ypT4 was an independent predictor of worse LRRFS (HR 15.14, *p* = 0.026). The 5-year LRRFS was significantly lower in TRG3–5 patients with a distal margin of ≤1 mm compared to those with a distal margin of >1 mm (68.5% vs. 84.2%, *p* = 0.027), while no difference was observed in case of TRG1–2 (*p* = 0.77). Conclusions: Low-responder rectal cancers after nCRT still require a distal margin of >1 mm to reduce the high likelihood of local relapse.

## 1. Introduction

A classic but still unsolved question in surgical oncology is the adequacy of the distal margin in rectal cancer [1,2,3]. Indeed, historical evidence has demonstrated that a narrow distal margin may impact the risk of local recurrence, mainly due to the risk of discontinuous distal spread of cancer [4,5]. Further evidence has greatly minimized the role of the distal margin in disease recurrence, and the adequate margin length has been shortened from 2 cm to 1 cm [6,7]. More recently, the need for a distal margin of at least 1 cm has been questioned, since no significant differences in local recurrence rate have been observed in patients with distal margins of 8 mm, 5 mm, 2 mm, and even 1 mm [8,9,10]. Thus, while an involved circumferential margin has been firmly associated to a higher risk of local recurrence and worse survival [11], the importance of the distal margin in rectal cancer remains controversial. In the last decade, the multimodal treatment of rectal cancer has been optimized, including the introduction of “total neoadjuvant therapy”, which has increased the complete or major response rate [12]. The addition of preoperative chemotherapy after short-course radiotherapy was recently assessed in the RAPIDO trial [13], while the escalation of neoadjuvant chemotherapy by administering FOLFIRINOX before long-course radiotherapy was evaluated in the PRODIGE 23 trial [14]: both studies demonstrated a significant increase in the pathologic complete response rates [15,16]. More recently, a complete response rate of 100% was evidenced in a cohort of patients with mismatch repair-deficient rectal cancer treated by neoadjuvant administration of the anti-PD1 dostarlimab [17]. Thus, updated evidence assessing the role of the distal margin in rectal cancer in current clinical practice would be highly desirable. Furthermore, the adequate length of the distal margin has implications of paramount relevance in terms of patients’ quality of life, since the acceptability of a closer margin might enable a higher rate of sphincter-saving rectal resections [18]. The aim of the present study was to assess the impact of a microscopically close or positive distal margin, defined as a clear margin length of ≤1 mm, on 5-year locoregional recurrence-free survival in rectal cancer treated by neoadjuvant therapy.

## 2. Patients and Methods

### 2.1. Study Population

All consecutive patients affected by rectal cancer and treated by neoadjuvant chemoradiotherapy (nCRT) and subsequent surgery at the Colorectal Surgery Unit of the National Cancer Institute of Milan, Italy, between 2014 and 2021 were collected from a prospectively maintained database, authorized by the Institutional Review Board of the Institute (protocol no. 149/2019). The inclusion criteria were a preoperative diagnosis of rectal cancer proven on endoscopic biopsy, indication for nCRT, extra-peritoneal localization of rectal cancer, and availability of margin status on the pathology report. Patients with a positive mesorectal (circumferential) margin, those treated by abdominoperineal resection or trans-anal local excision, localized in the upper rectum or at recto-sigmoid junction, with squamous cell carcinoma, or treated with palliative intent were excluded from the present study.

### 2.2. Study Design and Endpoints

Patients were divided into two groups: those with a distal margin of ≤1 mm vs. those with a distal margin of >1 mm. The distribution of baseline clinical and pathological variables between the two groups was verified to assess whether cohorts were balanced. Then, survival analyses were performed with Kaplan–Meier curves to assess the impact of ≤1 mm vs. >1 mm distal margins on the endpoints. The primary endpoint of the study was the 5-year locoregional recurrence-free survival (LRRFS), defined as the time between surgery and the occurrence of a pelvic mass or regional lymphadenopathy or any other clinical or radiological sign of local relapse, proven by biopsy or with imaging features highly suggestive of malignancy. The secondary endpoints were (1) the 5-year distant metastasis-free survival (DMFS), defined as the time between surgery and the occurrence of distant lesions, even if not proved on biopsy; (2) the 5-year disease-free survival (DFS), defined as the time between surgery and the occurrence of any first event, whether local or distant relapse. Then, univariate and multivariate survival analyses were performed to evaluate the independent predictive role of a distal margin of ≤1 mm on LRRFS in nCRT patients, accounting for all variables possibly related to the outcome.

### 2.3. Multidisciplinary Treatment of Rectal Cancer

All patients were evaluated at the weekly multidisciplinary Tumor Board of the Colorectal Surgery Unit after completion of staging by thoraco-abdominal computed tomography (CT) scan and pelvic magnetic resonance imaging (MRI). Based on the clinical T and N stage assessed on imaging, distance from the anal verge, threatened circumferential margins, age, and possible comorbidities, patients were proposed for nCRT. In case of standard long-course nCRT, patients received 825 mg/m^2^ BID of capecitabine and concurrent administration of radiotherapy (total dose: 54 Gy delivered in 25–27 fractions) targeting the rectal cancer and mesorectum. Then, surgery was performed about 8–12 weeks after nCRT completion. In case of short-course neoadjuvant radiotherapy, patients received a total dose of 25 Gy delivered in 5 fractions, with planning of surgery after 4–6 weeks. Patients with locally advanced rectal cancers (cT4 and/or cN2) were selected for total neoadjuvant therapy regimens, including systemic chemotherapy with XELOX/FOLFOX/FOLFIRI or FOLFOXIRI followed by standard nCRT. Some patients were accrued in prospective trials on neoadjuvant immunotherapy, and they received long-course chemoradiation with capecitabine and avelumab or durvalumab. Surgery was performed mainly via an open approach from 2017 to 2018, then mainly via a laparoscopic approach from 2019 to 2021. A stapled colorectal anastomosis was performed by trans-anal circular stapler for resections just above the anorectal junction; otherwise, a manual coloanal anastomosis was preferred. In almost all cases, a loop colostomy or ileostomy was also fashioned. The minimal distal margin to be achieved as the intraoperative goal was 10 mm, but after histopathological assessment, a distal margin of ≤1 mm was accepted in case of R0 resection. Conversely, cases with microscopically involved distal margins (R1) were addressed to completion abdomino-perineal resection, if accepted by the patients. Adjuvant treatment was planned based on final pathology and type of nCRT, according to guidelines. Follow-up was performed via thoraco-abdominal CT scan, colonoscopy, and CEA every 6 months for 5 years. Pelvic MRI could be added if locoregional relapse was suspected.

### 2.4. Statistical Analysis

Differences between patients with a distal margin of ≤1 mm vs. >1 mm were assessed to verify the heterogeneity of the study population. Continuous variables are reported as the mean ± standard deviation and were compared using a Student’s T test or non-parametric Wilcoxon test, as appropriate. Categorical variables are expressed as absolute numbers and percentages and were compared using a *χ*^2^ test or Fisher exact test. The impact of a distal margin of ≤1 mm on the endpoints was assessed via a Cox proportional hazards regression model, including variables significantly associated with the outcomes to avoid any bias. The LRRFS probabilities were estimated by the Kaplan–Meier method. Statistical significance was set at *p* < 0.05 (two-tailed). Data analysis was performed using GraphPad PRISM v. 9 (Dotmatics, Boston, MA, USA).

## 3. Results

### 3.1. Clinical Characteristics of Included Patients

**Figure 1:**
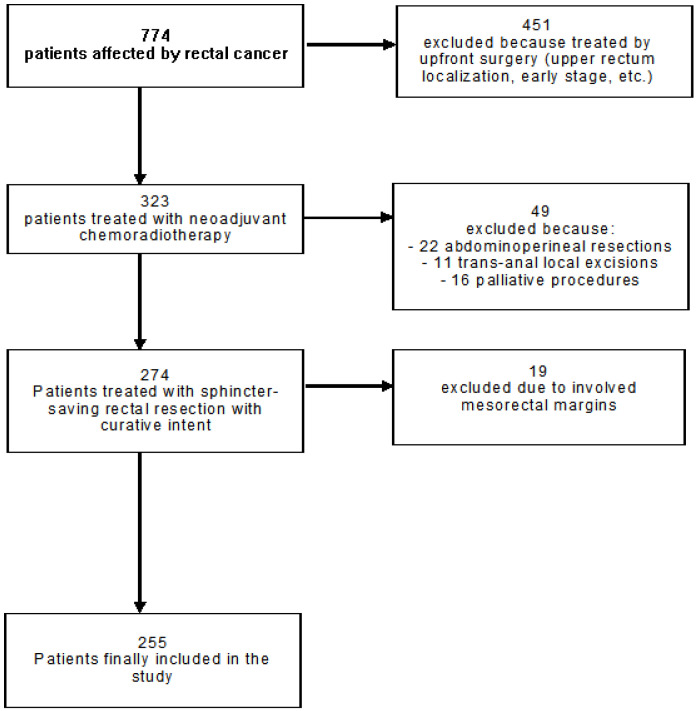
Flow chart of the included patients.

From a total of 774 patients affected by rectal cancer, 451 (58.3%) were excluded due to upper rectum/intraperitoneal localization or other indications for upfront surgery, 19 (2.5%) were excluded due to an involved mesorectal margin, 22 (2.8%) were excluded due to abdominoperineal resection, 11 (1.4%) were excluded due to trans-anal local excision, and 16 (2.1%) were excluded due to palliative surgery. Thus, 255 patients were included in the final analyses (Figure 1).

Patients were divided into two groups: 83 (32.5%) had a distal margin of ≤1 mm and 172 (67.5%) had a distal margin of >1 mm on final pathology. The baseline clinical and pathological characteristics between the two groups were balanced, except for a lower mean distance of cancer from the anal verge (3.9 ± 2.3 vs. 6.1 ± 2.7 cm, *p* < 0.0001) in patients with a distal margin of ≤1 mm. The mean numbers of harvested locoregional lymph nodes were similar between the groups (14.6 ± 6.0 vs. 15.1 ± 7.7, *p* = 0.604). Additionally, the neoadjuvant rectal (NAR) score was not significantly different between the groups (*p* = 0.205) [19]. All other features are reported in Table 1.

### 3.2. Type of Neoadjuvant Treatment

The most frequent type of neoadjuvant treatment was standard long-course chemoradiation (199 patients, 78.0%), followed by short-course radiotherapy (32, 12.5%), long-course nCRT plus immunotherapy within clinical trials (17, 6.67%), and total neoadjuvant therapy (7, 2.7%). No difference in the distribution of nCRT type in distal margins of ≤1 mm vs. >1 mm was observed (*p* = 0.51), as reported in Table 1.

### 3.3. Survival Analyses

**Figure 2:**
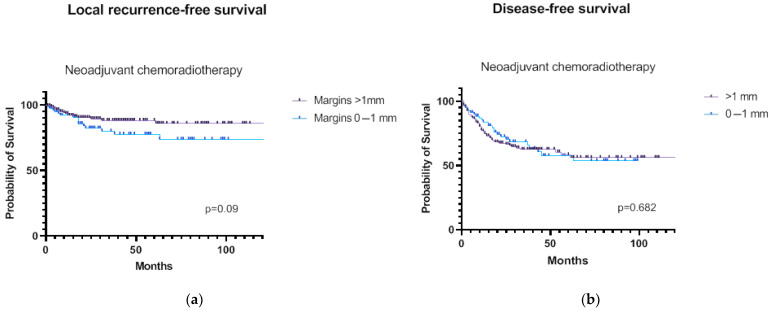
Survival curves of rectal cancer patients with a distal margin of ≤1 mm vs. >1 mm: (**a**) locoregional recurrence-free survival; (**b**) Disease-free survival.

The included patients had a median follow-up time of 42.1 months (interquartile range: 14–63 months). Crude locoregional recurrence rates were 18.1% in patients with a distal margin of ≤1 mm vs. 9.9% in patients with a distal margin of >1 mm (*p* = 0.064). The 5-year LRRFS rate was 77.6% with a distal margin of ≤1 mm vs. 88.3% with a distal margin of >1 mm (Log-rank *p* = 0.09, Figure 2a). The 5-year DMFS rates were, respectively, 72.1% vs. 64.8% (Log-rank *p* = 0.11), and the 5-year DFS rates were 57.6% vs. 58.4% (Log-rank *p* = 0.682, Figure 2b). Among cases with a distal margin of ≤1 mm, patients with a microscopically positive margin (R1 resection) had a lower 5-year LRRFS compared to patients with a close but clear distal margin (R0 resection), being 65.1% vs. 81.9% (Log-rank *p* = 0.037, Supplementary Figure S1). Furthermore, the 5-year LRRFS of patients treated with standard fluoropyrimide-based neoadjuvant chemoradiation was similar to that of patients treated with other neoadjuvant regimens (Log-rank *p* = 0.275, Supplementary Figure S2).

### 3.4. Predictors of LRRFS in Patients Treated by nCRT

On univariate Cox analysis, stage ypT4 (HR 20.08, 95%CI 5.13–132.4, *p* = 0.0001), category ypN2 (HR 1.19, 95%CI 0.28–3.58, *p* = 0.052), and TRG3–5 (HR 2.90, 95%CI 1.32–7.28, *p* = 0.013) were predictive of worse LRRFS in nCRT patients. On multivariate analysis, only stage ypT4 remained an independent predictor of worse LRRFS (HR 9.25, 95%CI 1.31–83.92, *p* = 0.03). The univariate and multivariate analyses are reported in Table 2.

### 3.5. Correlation between Distal Margins, Tumor Regression Grade, and LRRFS in nCRT Patients

**Figure 3:**
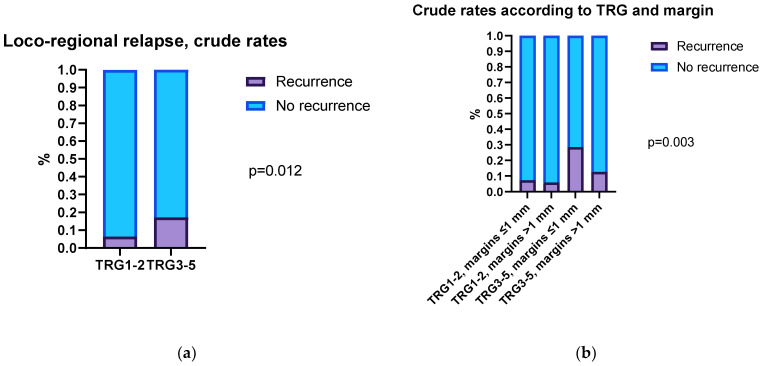
Association between locoregional recurrence, tumor regression grade, and distal margin in rectal cancer patients treated by neoadjuvant chemoradiotherapy: (**a**) crude rates of locoregional relapse according to different tumor regression grades; (**b**) crude rates of locoregional relapse according to different tumor regression grades and distal margins.

The correlation between TRG and locoregional relapse was assessed. A trend toward increased crude recurrence rates in partial/low responders was observed, being 4.9% in TRG1, 7.2% in TRG2, 15.3% in TRG3, and 20.0% in TRG4–5 patients (*p* = 0.06). The locoregional recurrence rate was higher in patients with TRG3–5 compared to TRG1–2 (*p* = 0.012, Figure 3a). Then, a correlation analysis was performed by stratifying patients according to TRG and distal margins of >1 mm vs. ≤1 mm. Patients with a TRG of 3–5 and a distal margin of ≤1 mm showed a higher local recurrence rate (28.6%) compared to the other groups (*p* = 0.003, Figure 3b).

**Figure 4:**
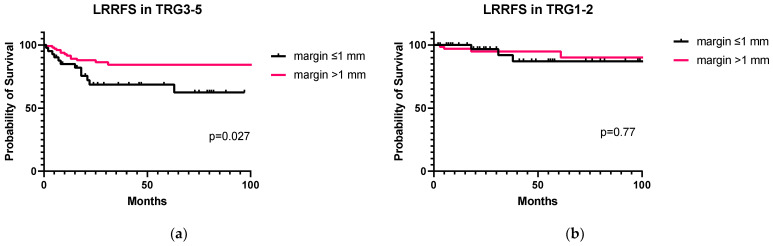
Locoregional recurrence-free survival curves of rectal cancer patients with a distal margin of ≤1 mm vs. >1 mm: (**a**) patients with a TRG (Mandard) of 3–5; (**b**) Patients with a TRG (Mandard) of 1–2.

The 5-year LRRFS rate was significantly lower in TRG3–5 patients with a distal margin of ≤1 mm compared to those with a distal margin of >1 mm (68.5% vs. 84.2%, Log-rank *p* = 0.027, Figure 4a), while no difference was observed in patients with a distal margin of ≤1 mm vs. >1 mm in case of TRG1–2 (87.0% vs. 94.7%, Log-rank *p* = 0.77, Figure 4b). Finally, the impact of adjuvant chemotherapy in nCRT patients with TRG3–5 and a distal margin of ≤1 mm was assessed: the 5-year LRRFS was similar in patients treated with adjuvant chemotherapy and in those who were not, at 75.5% vs. 82.6%, respectively (Log-rank *p* = 0.971).

## 4. Discussion

The adequacy of the distal margin in rectal cancer is still controversial, particularly in the rapidly evolving world of available neoadjuvant treatments, considering the risk of distal intramural or mesorectal spread after nCRT [20]. Escalation of chemotherapy within neoadjuvant therapy toward a “total neoadjuvant treatment” and the recent introduction of neoadjuvant immunotherapy in MSI-H rectal cancer are strongly increasing the pCR and major response rates [17]. These promising findings could pave the way to minimize the extent of the clear distal margin, increasing the likelihood of an ultralow colorectal or coloanal anastomosis and, thus, the possibility of sphincter-saving surgery.

The present study highlights that a pathologic finding of a distal margin of ≤1 mm is not related to lower 5-year LRRFS in patients with a major response or pCR (87.0% vs. 94.7%, *p* = 0.77), provided that R0 resection is achieved [21], but a worse LRRFS was observed with a distal margin of ≤1 mm in TRG3–5 patients (68.5% vs. 84.2%, *p* = 0.027). In other words, a ≤1 mm clear margin width seems to be inadequate in low responders, probably due to the overwhelmingly high risk of residual tumor burden beyond the distal edge of the tumor [22,23,24]. Indeed, a distal margin of ≤1 mm in low responders means not only a higher risk of mucosal/submucosal spread of tumor over the resection edge, but also a higher likelihood of incomplete mesorectal excision with possible residual nodal disease [25], despite similar mean numbers of harvested nodes being observed with close vs. clear margins (14.6 vs. 15.1, *p* = 0.604). Furthermore, patients treated with nCRT more often had low or ultralow rectal cancer, and coloanal anastomosis was more frequently performed. Thus, the distal edge of rectal resection represented the true distal margin in these patients, since no anastomotic “doughnut” was available as in stapled circular end-to-end colorectal anastomoses [26]. Moreover, in low responders, the residual rectal cancer after nCRT is expected to constitute chemo/radio-resistant cells, thus having a more aggressive biology and probably a higher risk to locally recur [27]. Indeed, adding adjuvant chemotherapy in these patients did not minimize the detrimental effect of a close distal margin on 5-year LRRFS (75.5% vs. 82.6%, *p* = 0.971), further highlighting that residual rectal cancer might be chemo-refractory and emphasizing the importance of radical surgery.

On multivariate analyses, the distal margin was not an independent predictor of local recurrence (HR 1.87, *p* = 0.092). Only stage (y)pT4 was significantly associated with a worse LRRFS (HR 9.25, *p* = 0.03), due to the higher likelihood of residual disease over the mesorectal fascia [28]. In other words, the circumferential margin is much more relevant and predictive of LRRFS than the distal margin, despite patients with a positive mesorectal margin being excluded from the study [29]. Not surprisingly, the great majority of local relapses from rectal cancer frequently occur laterally, anteriorly, or posteriorly into the pelvis, while central/luminal recurrences (mostly depending on the distal margin) have become much more uncommon in recent years [30].

Several previous small series have explored the role of a close/microscopically positive distal margin on survival rates, with controversial findings. Some authors have demonstrated that a close distal margin does not affect survival in rectal cancer patients, but mainly in cases treated by upfront surgery [31]. A possible explanation is that rectal cancer treated by immediate resection does not have peripheral areas of regression; thus, the microscopic distal edge of the lesion probably represents the true distal border, with a low risk of tumor spread over the distal margin [32].

A precedent study from our institution demonstrated that 5-year overall survival was significantly lower in patients with a microscopically positive distal margin compared to patients with a <1 cm or ≥1 cm margin, at rates of 51%, 81%, and 69%, respectively (*p* = 0.018) [33]. However, most of the included patients were treated before 2000, when principles of a proper total mesorectal excision and nCRT were still not widely adopted. Other authors have reported an increased risk of mucosal and pelvic relapse with a distal margin of <8 mm, but, interestingly, the strength of association on multivariate analysis (HR 1.10, *p* = 0.01) was not as high as that with other features such as nodal involvement (HR 5.25, *p* = 0.0006) or the presence of distant metastases (HR 25.06, *p* < 0.0001) [8]. More recently, a large cohort study demonstrated a higher 5-year local recurrence rate in patients with a distal margin of ≤1 mm vs. >1 mm (24.1% vs. 12.0%, *p* = 0.005), but a significant improvement in local relapse rate was observed simply by adding 1 mm of clear margin (22.7% with a distal margin of ≤1 mm vs. 12.4% with ≤2 mm, *p* = 0.035), and no separate analyses were performed for patients treated with upfront surgery vs. nCRT [10]. Other authors observed no difference in LRRFS between close and clear distal margins (HR 1.1, *p* = 0.29) specifically in patients treated with nCRT [34].

The present study has limitations and controversial findings. A very high rate of patients with a distal margin of ≤1 mm was reported, at 32.5%, despite current guidelines recommending a distal margin of at least 5 mm. It should be noted that the great majority of included patients had low/ultralow rectal cancer, with a mean distance from the anal verge of 3.9–6.1 cm. Moreover, the included patients had mostly locally advanced rectal cancer at diagnosis, with a clinical stage of cT3–T4 in 91.0% and cN1–N2 in 83.5% of cases. After surgery, only patients with a microscopically positive margin (R1) were candidates for a re-intervention (completion abdomino-perineal resection), further explaining the high rate of close distal margins in the cohort of patients.

Secondly, although a distal margin of ≤1 mm was expected to be associated to a higher incidence of locoregional recurrences, the 5-year LRRFS of 77.6% was overwhelmingly low. However, some of these patients had microscopically positive margins, and R1 resection probably had the greatest impact on LRRFS, since the 5-year LRRFS rate was 65.1% in R1 cases but 81.9% in R0 patients. An R1 resection means that viable cancer cells might remain after surgery on the distal rectal stump, and even a proper multimodal treatment cannot minimize the risk of these local relapses.

Third, a 4.9% relapse rate was observed even in patients with Mandard TRG1. Other reports have described a local and/or distant recurrence rate of up to 9.2% in rectal cancer patients despite pathologic complete response [35]. Furthermore, three patients with a Mandard TRG1 observed on the primary tumor site had one or more positive locoregional lymph nodes, potentially explaining this finding.

## 5. Conclusions

The intraoperative goal and the pathologic finding of the distal margin are profoundly different features. Whenever possible, the surgeon should try to achieve a macroscopically safe distal margin to minimize the risk of local relapse. This intraoperative goal should be at least 1 cm, as recommended by current guidelines [36], to reduce the likelihood of an inadequate pathologic distal margin. However, in the current standard-of-care practice, if a major response is observed after neoadjuvant chemoradiation, a pathologic finding of a distal margin of ≤1 mm might be acceptable and a second surgery (often abdominoperineal resection) could be avoided, provided that R0 resection is achieved. Low-responder rectal cancers, being the expression of residual chemo/radio-resistant cells, still require a distal margin of >1 mm to reduce the high likelihood of local relapse.

## Figures and Tables

**Table 1:** Clinical and pathological features between patients with a distal margin of ≤1 mm vs. >1 mm.

	≤1 mm (*n* = 83)	>1 mm (*n* = 172)	*p* Value
**Gender**			0.247
Male	55 (66.3%)	101 (58.7%)	
Female	28 (33.7%)	71 (41.3%)	
**ASA Score**			0.544
ASA I–II	65 (87.8%)	124 (90.5%)	
ASA III	9 (12.2%)	13 (9.5%)	
Data missing	9	35	
**Age**	59.9 ± 12.3	60.1 ± 11.1	0.897
**CEA**	6.3 ± 33.4	5.8 ± 9.8	0.856
**CA 19.9**	12.6 ± 11.4	15.2 ± 15.5	0.175
**cT category**			0.886
cT1	3 (3.6%)	3 (1.7%)	
cT2	6 (7.2%)	11 (6.4%)	
cT3a–b	35 (42.2%)	76 (44.2%)	
cT3c–d	30 (36.2%)	66 (38.4%)	
cT4	9 (10.8%)	16 (9.3%)	
**cN category**			0.022
cN0	21 (25.3%)	21 (12.2%)	
cN1	43 (51.8%)	113 (65.7%)	
cN2	19 (22.9%)	38 (22.1%)	
**Distance from anal verge (cm)**	3.9 ± 2.3	6.1 ± 2.7	<0.0001
**Type of neoadjuvant treatment**			0.51
Long-course nCRT	68 (81.9%)	131 (76.2%)	
Short-course radiotherapy	7 (8.5%)	25 (14.5%)	
Total neoadjuvant therapy	3 (3.6%)	4 (2.3%)	
nCRT + Immunotherapy	5 (6.0%)	12 (7.0%)	
**ypT category**			0.226
ypT0	14 (16.9%)	27 (15.7%)	
ypT1–2	33 (39.8%)	51 (29.7%)	
ypT3	30 (36.1%)	85 (49.4%)	
ypT4	6 (7.2%)	9 (5.2%)	
**ypN stage**			0.266
ypN0	58 (69.9%)	108 (62.8%)	
ypN+	25 (30.1%)	64 (37.2%)	
**Harvested lymph nodes**	14.6 ± 6.0	15.1 ± 7.7	0.604
**TRG (Mandard)**			0.502
TRG1	14 (16.9%)	27 (15.7%)	
TRG2	27 (32.5%)	42 (24.4%)	
TRG3	26 (31.3%)	59 (34.3%)	
TRG4	15 (18.1%)	37 (21.5%)	
TRG5	1 (1.2%)	7 (4.1%)	
**pCR**			0.54
Yes	14 (16.9%)	24 (14.0%)	
No	69 (83.1%)	148 (86.0%)	
**NAR score**			0.205
NAR < 8	16 (19.3%)	30 (17.4%)	
NAR 8–16	44 (53.0%)	75 (43.6%)	
NAR > 16	23 (27.7%)	67 (39.0%)	
**Anastomotic leak**			0.297
Yes	13 (15.7%)	19 (11.0%)	
No	70 (84.3%)	153 (89.0%)	
**Adiuvant chemotherapy**			0.109
Yes	44 (53.0%)	95 (63.8%)	
No	39 (47.0%)	54 (36.2%)	
Data missing	8	23	

**Table 2:** Univariate and multivariate analyses for LRRFS in patients treated by neoadjuvant chemoradiation.

	Univariate Analysis			Multivariate Analysis		
	HR	95%CI	*p* Value	HR	95%CI	*p* Value
**Age**	0.98	0.96–1.01	0.267			
**Gender**	1.01	0.50–2.10	0.971			
**CEA**	1.00	0.99–1.01	0.165			
**Distance from the anal verge**	0.95	0.84–1.06	0.377			
**Short course vs. long course**	2.27	0.82–5.42	0.083			
**Laparoscopy vs. laparotomy**	0.27	0.015–1.29	0.204			
**Anastomotic leakage**	1.32	0.45–3.16	0.569			
**ypT category**						
ypT0	(ref)	(ref)	(ref)	(ref)	(ref)	(ref)
ypT1	3.41	0.41–28.41	0.221	2.48	0.29–21.24	0.372
ypT2	1.84	0.39–12.84	0.468	1.06	0.17–8.44	0.949
ypT3	2.95	0.82–18.77	0.153	1.50	0.26–11.9	0.664
ypT4	20.08	5.13–132.4	0.0001	9.25	1.31–83.92	0.003
**ypN stage**						
ypN0	(ref)	(ref)	(ref)	(ref)	(ref)	(ref)
ypN1	2.07	0.98–4.29	0.782	1.08	0.44–2.52	0.869
ypN2	1.19	0.28–3.58	0.052	0.77	0.17–2.59	0.702
**Mandard TRG**						
TRG1–2	(ref)	(ref)	(ref)	(ref)	(ref)	(ref)
TRG3–5	2.90	1.32–7.28	0.013	2.30	0.83–7.91	0.143
**Distal margin ≤ 1 mm**	1.81	0.89–3.63	0.094	1.87	0.89–3.87	0.092
**MSI-H/dMMR**	2.59	0.40–9.49	0.213			
**Adjuvant chemotherapy**	0.87	0.42–1.89	0.708			

## Data Availability

Data are available from corresponding author upon request.

## Supplementary Materials

The following supporting information can be downloaded at https://www.mdpi.com/article/10.3390/cancers15061828/s1, Figure S1: Five-year LRRFS in patients with a microscopically positive distal margin vs. patients with a ≤1 mm clear distal margin; Figure S2: Five-year LRRFS in patients treated with standard fluoropyrimidine-based neoadjuvant chemoradiation vs. other regimens.
