# Supplementary material for: Impact of Microscopically Positive (≤1 mm) Distal Margins on Disease Recurrence in Rectal Cancer Treated by Neoadjuvant Chemoradiotherapy
Source: Cancers (Basel). 2023 Mar 17;15(6):1828. doi: 10.3390/cancers15061828 (PMC10047023; doi:10.3390/cancers15061828)
Supplement: Supplementary file 1 [file cancers-15-01828-s001.zip › cancers-2244087-supplementary.pdf]

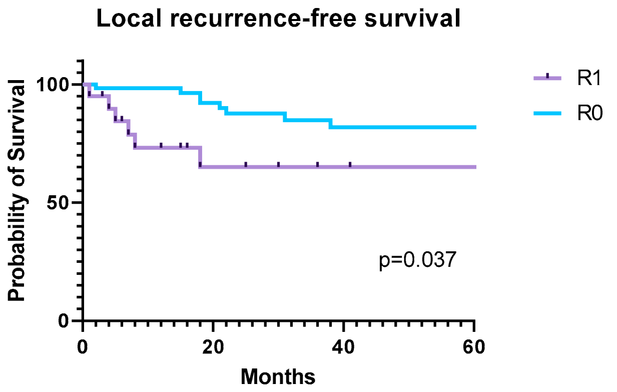

Figure S1. 5-year LRRFS in patients with a microscopically positive distal margin vs. patients with a ≤1 mm clear distal margin.

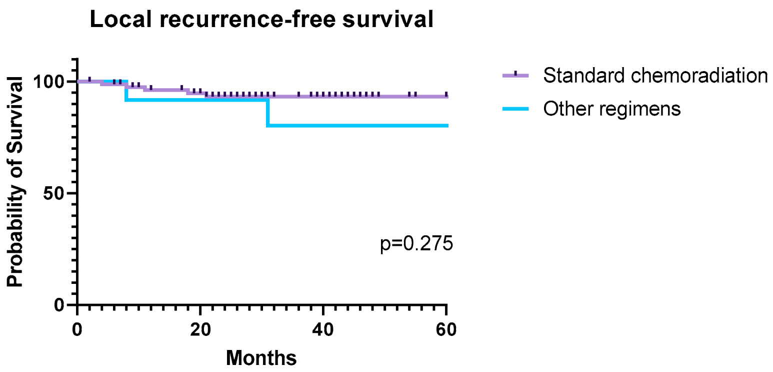

Figure S2. 5-year LRRFS in patients treated with standard fluoropyrimidine-based neoadjuvant chemoradiation vs. other regimens.
